# 2023 European Society of Cardiology guidelines for the management of acute coronary syndromes: Statement of endorsement by the NVVC

**DOI:** 10.1007/s12471-024-01896-2

**Published:** 2024-09-10

**Authors:** Bastiaan Zwart, Bimmer E. P. M. Claessen, Peter Damman, Pier Woudstra, Maarten A. Vink, J. Willem Balder, Michael G. Dickinson, Erik A. Badings, Yolande Appelman, Arnoud W. J. van ’t Hof, Jurriën M. ten Berg, Fatih Arslan

**Affiliations:** 1 University Medical Centre Groningen, Groningen, The Netherlands; 2 Amsterdam University Medical Centre, location AMC, Amsterdam, The Netherlands; 3 Radboud University Medical Centre, Nijmegen, The Netherlands; 4 Medisch Centrum Leeuwarden, Leeuwarden, The Netherlands; 5 Onze Lieve Vrouwe Gasthuis, Amsterdam, The Netherlands; 6 University Medical Centre Utrecht, Utrecht, The Netherlands; 7 Deventer Ziekenhuis, Deventer, The Netherlands; 8 Amsterdam UMC, location VUmc, Amsterdam, The Netherlands; 9 Maastricht University Medical Centre, Maastricht, The Netherlands; 10 Zuyderland MC, Heerlen, The Netherlands; 11 St. Antonius Hospital, Nieuwegein, The Netherlands; 12 Leiden University Medical Centre, Leiden, The Netherlands

**Keywords:** Acute coronary syndrome, Antiplatelet therapy, Percutaneous coronary intervention, Guidelines

## Abstract

This review discusses the new recommendations in the 2023 European Society of Cardiology guidelines on the management of acute coronary syndrome and provides a perspective on topics specific to clinical practice in the Netherlands, including pre-treatment, antiplatelet agent strategies, the use of risk scores and logistical considerations with regard to the timing of coronary angiography.

## Introduction

The 2023 European Society of Cardiology (ESC) guidelines for the management of acute coronary syndrome (ACS) combine previously separate guidelines for ST-elevation myocardial infarction (STEMI) and non-ST-elevation acute coronary syndrome (NSTE-ACS), which date from 2017 and 2020, respectively. The document now covers the entire spectrum of ACS, ranging from unstable angina to STEMI [1].

The ACS Working Group was asked by the Netherlands Society of Cardiology (NVVC) to review the new guidelines and provide a Dutch perspective and critical appraisal that is relevant for the Dutch healthcare system and daily clinical practice. For several clinical settings, we describe how and why Dutch practice might deviate from the 2023 ESC guidelines. Differences between the 2023 ESC guidelines and the Dutch ACS Working Group recommendations are summarised in Tab. 1.

## Pre-treatment

Antiplatelet therapy serves as the cornerstone of pharmacological intervention in patients with coronary artery disease, particularly those presenting with ACS. This therapeutic approach is aimed at reducing the occurrence of stent and atherosclerotic disease-related ischaemic events while taking into account the bleeding risk of the patient.

### P2Y_12_ inhibitor pre-treatment in STEMI

In the Netherlands, patients with STEMI eligible for primary percutaneous coronary intervention (pPCI) are pre-treated with a loading dose of ticagrelor/prasugrel, aspirin and unfractionated heparin by paramedics in the ambulance. However, pre-treatment with a P2Y_12_ inhibitor received merely a class IIb, level of evidence (LoE) B recommendation in the 2023 ESC guidelines. From our perspective, the use of pre-hospital ticagrelor/prasugrel in STEMI patients, following the design of the PLATO and TRITON trials, appears reasonable given current health care logistics in the Netherlands (with very high use of the radial approach and therefore a lower bleeding risk) and is supported by contemporary evidence that pre-treatment with ticagrelor/prasugrel is a safe approach for these patients.

So far, only the ATLANTIC trial has investigated the different timing regimens of ticagrelor pre-treatment, namely pre-hospital versus in-hospital before pPCI, in STEMI patients [2]. No differences were found in ST resolution or TIMI III flow prior to pPCI, nor in the composite endpoint of mortality, MI, stroke, urgent coronary revascularisations, or stent thrombosis. However, pre-hospital administration of ticagrelor did reduce the occurrence of definite stent thrombosis at 24 h (0% vs 0.8%, *p* = 0.008) and 30 days (0.2% vs 1.2%; *p* = 0.02). It is noteworthy that the median time difference between the two timing strategies was only 31 min. Bleeding rates were comparable between the two treatment groups. A comprehensive meta-analysis, encompassing three randomised controlled trials (RCTs) and 14 observational studies, indicated that pre-treatment was not associated with a reduction of overall cardiovascular events. However, within the subgroup of patients receiving pre-hospital P2Y_12_ inhibition, there was a decreased 30-day risk of MI [3]. In conclusion, there is no evidence that pre-hospital P2Y_12_ inhibition increases reperfusion, but it does reduce post-PCI thrombotic complications such as stent thrombosis. Currently, the effect of pre-treatment with subcutaneous formulation of novel drugs is being studied as regards the outcome in STEMI patients (CELEBRATE trial, NCT04825743; SOS-AMI trial, NCT04957719).

### Anticoagulation pre-treatment in STEMI

Unfractionated heparin is recommended as standard of care for patients with STE-ACS and is most often given in the ambulance during transfer to a PCI centre. Fondaparinux (a selective Xa inhibitor) use is not recommended in STEMI patients undergoing pPCI, based on the results of the OASIS-6 trial [4].

### P2Y_12_ inhibitor pre-treatment in NSTE-ACS

The 2023 guidelines state that pre-treatment with a P2Y_12_ inhibitor is not recommended when the coronary anatomy is unknown (class III, LoE A) and when early (< 24 h) invasive management is planned. This change in recommendation first appeared in the 2020 guidelines following the ISAR-REACT-5 study, demonstrating that prasugrel with no pre-loading was superior to ticagrelor in the context of pre-loading [5]. However, it must be noted that coronary angiography in the NSTE-ACS group was performed within a mean of 4 h after randomisation (personal communication with investigators from the ISAR group), which is not representative for Dutch clinical practice.

The guidelines do offer room for pre-treatment, stating that pre-treatment with P2Y_12_ inhibitors may be considered (class IIb, LoE C) in patients who cannot undergo an early invasive strategy and who do not exhibit a high bleeding risk, as defined by the Academic Research Consortium on High Bleeding Risk (ARC-HBR) criteria [6].

Several other aspects need to be taken into account when deciding on pre-treatment. Pre-treatment with a P2Y_12_ inhibitor on top of aspirin and low-molecular-weight heparin imposes a substantial risk of bleeding, especially in older patients or in patients otherwise at high bleeding risk. Second, in patients proceeding to coronary artery bypass grafting the surgical risk is significantly increased. On the other hand, bleeding risk in the Netherlands (with radial access being used in a very high proportion of cases) might be lower than in previous studies investigating the benefits and risks of pre-treatment. Of note is that in the Comparison of Prasugrel at the Time of Percutaneous Coronary Intervention (PCI) or as Pretreatment at the Time of Diagnosis in Patients with Non-ST Elevation Myocardial Infarction (ACCOAST) study, in which pre-treatment resulted in significantly more bleeding, over 50% of patients underwent angiography via femoral access.

Overall, the ACS Working Group supports the class III recommendation not to use pre-treatment routinely. If coronary angiography cannot be performed within 24 h for logistical reasons, it is reasonable to pre-treat those patients deemed to be at high ischaemic risk and low bleeding risk with a P2Y_12_ inhibitor while they await angiography [1, 7].

### Choice of P2Y_12_ inhibitor in ACS

The guidelines support the use of a more potent P2Y_12_ inhibitor and advise the use of clopidogrel only when prasugrel and ticagrelor are not available. In older patients, clopidogrel may be considered (class IIb, LoE B), based on the POPULAR-AGE trial [8]. With respect to which potent P2Y_12_ inhibitor to choose, the guidelines state that prasugrel should be considered in preference to ticagrelor for ACS patients who proceed to PCI (class IIa, LoE B).

However, this preference for prasugrel is based on limited evidence, as we previously set out [9]. The ISAR-REACT 5 trial is the only study comparing two antiplatelet strategies (pre-treatment with ticagrelor vs treatment with prasugrel post-PCI) rather than a head-to-head-comparison [5].

The ACS Working Group supports the use of both prasugrel or ticagrelor in NSTE-ACS patients. Factors to be taken into account when choosing a P2Y_12_ receptor inhibitor include logistical and patient- and drug-related considerations (dosing frequency, side effects, prasugrel contraindicated in stroke patients and no net benefit in the elderly). Of note is that prasugrel should not be used for pre-treatment.

### Anticoagulation in NSTE-ACS

Unfractionated heparin is recommended as standard care for patients with NSTE-ACS during invasive angiography or PCI. In patients not at high bleeding risk who are not undergoing immediate invasive treatment, fondaparinux therapy is recommended while they await angiography, based on the results of the OASIS-5 trial [10].

## Antithrombotic therapy and use of risk scores

### Antithrombotic therapy

The 2023 ESC guidelines encourage individualised antithrombotic treatment. Although the default strategy of 12 months of dual antiplatelet therapy (DAPT) with aspirin and a strong P2Y_12_ inhibitor (prasugrel or ticagrelor) remains unchanged, the guidelines offer guidance on specific clinical scenarios in which the default DAPT duration can be shortened (< 12 months), extended (> 12 months) or modified (de-escalation: i.e. switching from a strong P2Y_12_ inhibitor to clopidogrel).

In recent years, advancements in stent technology and intracoronary imaging-guided PCI have significantly reduced the risk of stent thrombosis. On the other hand, bleeding events carry a high risk of morbidity and mortality [11]. Therefore, emphasis should be shifted to tailoring treatment based on ischaemic and bleeding risk. The new guidelines advise that single antiplatelet therapy (SAPT) be considered after 3–6 months in patients who are event-free and are not at high ischaemic risk (class IIa, LoE A). If so, SAPT with a P2Y_12_ inhibitor should be preferred. In patients at high bleeding risk, a SAPT strategy may be considered even after 1 month (class IIb, LoE B). The LEGACY trial is currently evaluating the safety and efficacy of even earlier omittance of aspirin, i.e. immediate P2Y12 monotherapy, in NSTE-ACS patients [12].

In the elderly, especially those at high bleeding risk, the option of starting with clopidogrel instead of a strong P2Y_12_ inhibitor in order to reduce the bleeding risk may be considered (class IIb, LoE B).

Furthermore, P2Y_12_ inhibitor de-escalation in ACS patients may be considered as an alternative strategy to the default 12-month regimen, in order to reduce the risk of bleeding events. The guidelines do not recommend de-escalation in the first 30 days after an ACS due to a potentially increased risk of ischaemic events (class III, LoE B).

Interestingly, the POPULAR Genetics trial demonstrated that an early (in-hospital) CYP2C19 genotype-guided de-escalation strategy switching from ticagrelor to clopidogrel reduced bleeding while being non-inferior to standard treatment with respect to thrombotic events [13]. Moreover, this genotype-based strategy led to a significant cost reduction when applied in the Netherlands [14]. As CYP2C19 genotyping is now more widely used and available in many Dutch hospitals, efforts should be made to incorporate genotype-based strategies into clinical practice, especially for ACS patients at high bleeding risk.

### Triple therapy

In ACS patients with an indication for oral anticoagulation (non-vitamin‑K oral anticoagulant preferred to vitamin‑K antagonist), triple antithrombotic therapy (TAT) with the addition of aspirin and clopidogrel is recommended for up to 1 week, followed by 12 months of dual antithrombotic therapy (DAT; oral anticoagulant plus clopidogrel (class I, LoE A)). Generally, TAT is associated with a two- to threefold increase in bleeding risk [15] and therefore should be kept as short as possible. However, in patients with high ischaemic risk or other anatomical/procedural characteristics that outweigh the bleeding risk, TAT could be prolonged for up to 1 month (class IIa, LoE C). In patients with a very high risk of bleeding, maintenance DAT could be reduced to 6 months (class IIb, LoE B).

In medically managed ACS patients, current data support DAT over TAT, with a single antiplatelet agent (most commonly clopidogrel) for at least 6 months (class IIa, LoE B).

### Risk scores

As illustrated by the previous scenarios, patient selection is key. Several risk scores have been developed to identify patients at high risk for bleeding or thrombotic events during follow-up. However, all risk scores are hampered by poor discriminatory abilities.

The 2023 guidelines recommend using either the ARC-HBR criteria or the PRECISE-DAPT (PREdicting bleeding Complications in patients undergoing Stent implantation and subsEquent Dual Anti Platelet Therapy) score for the assessment of bleeding risk. The presence of one major or two minor ARC-HBR risk factors indicates a high bleeding risk (Tab. 2; [6]). Of note is that the presence of multiple major risk factors is associated with a progressive increase in the bleeding risk. The PRECISE-DAPT is an easier-to-use tool with only five parameters (haemoglobin, age, leukocyte count, creatinine clearance and prior bleeding). A PRECISE-DAPT score of ≥ 25 is regarded as a high bleeding risk [16].

**Table 1:** Differences between the 2023 European Society of Cardiology (*ESC*) guidelines for the management of acute coronary syndromes (*ACS*) and the Dutch ACS Working Group endorsement paper

		**ESC guidelines**	**Dutch ACS Working Group recommendations**
*Pre-treatment*	STEMI	Pre-treatment with a P2Y_12_ receptor inhibitor may be considered in STEMI patients undergoing a primary PCI strategy	Pre-treatment with a P2Y_12_ receptor inhibitor in STEMI patients undergoing primary PCI is reasonable given current logistics of the health care system in the Netherlands
	NSTE-ACS	Routine pre-treatment with a P2Y_12_ receptor inhibitor in NSTE-ACS patients in whom coronary anatomy is not known and early invasive management (< 24 h) is planned is not recommended	Routine pre-treatment in NSTE-ACS is not recommended. In patients in whom coronary angiography cannot be performed within 24 h for logistical reasons and who are deemed to be at high ischaemic risk and low bleeding risk, it is reasonable to pre-treat with a P2Y_12_ inhibitor while awaiting angiography
		Pre-treatment with a P2Y_12_ receptor inhibitor may be considered in NSTE-ACS patients who are not expected to undergo an early invasive strategy	
*Antiplatelet strategies*	Choice of antiplatelet agent	Prasugrel should be considered in preference to ticagrelor for ACS patients who proceed to PCI	The use of both prasugrel or ticagrelor is recommended for patients who proceed to PCI
	DAPT duration	In specific clinical scenarios, the default DAPT duration can be shortened (12 months) or modified (switching DAPT, DAPT de-escalation). The use of risk scores is recommended	Both bleeding risk and ischaemic risk should be assessed in a structured manner (using ARC-HBR or PRECISE-DAPT score for HBR pre-discharge and DAPT score for ischaemic risk)
			The interventional cardiologist should take the leading role in highlighting any high-risk features of recurrent ischaemic events related to the PCI or coronary anatomy
	De-escalation	De-escalation of P2Y_12_ receptor inhibitor treatment (e.g. with a switch from prasugrel/ticagrelor to clopidogrel) may be considered as an alternative DAPT strategy to reduce the bleeding risk	De-escalation strategies are encouraged. Specifically, the use of a CYP2C19-genotype-guided de-escalation strategy is recommended
*Logistical considerations*	Timing of coronary angiography	An early invasive strategy within 24 h should be considered in patients with a confirmed diagnosis of NSTE-ACS	An early invasive strategy (< 24 h) is recommended, specifically in patients with a GRACE risk score >140. If this is not possible from a logistical perspective, a delayed invasive strategy (< 72 h) is acceptable and safe
	Routing of patients with OHCA	Transport of patients with out-of-hospital cardiac arrest to a cardiac arrest centre according to local protocol should be considered	We advise that current regional arrangements for haemodynamically unstable patients without STEMI not be changed

*STEMI* ST-elevation myocardial infarction, *PCI* percutaneous coronary intervention, *NSTE-ACS* non-ST-elevation acute coronary syndrome, *DAPT* dual antiplatelet therapy, *ARC-HBR* Academic Research Consortium on High Bleeding Risk, *PRECISE-DAPT* PREdicting bleeding Complications in patients undergoing stent Implantation and SubsequEnt Dual Anti Platelet Therapy, *OHCA* out-of-hospital cardiac arrest

**Table 2:** Major and minor criteria for high bleeding risk at the time of percutaneous coronary intervention (*PCI*) according to the Academic Research Consortium

**Major**	**Minor**
	Age ≥ 75 years
Anticipated use of long-term oral anticoagulation	–
Severe or end-stage CKD (eGFR < 30 ml/min)	Moderate CKD (eGFR 30–59 ml/min)
Haemoglobin < 11 g/dl (< 6.8 mmol/l)	Haemoglobin 6.8–8.0 (mmol/l) for men and 6.8–7.4 (mmol/l) for women
Spontaneous bleeding requiring hospitalisation or transfusion within the past 6 months or at any time, if recurrent	Spontaneous bleeding requiring hospitalisation or transfusion within the past 12 months and not meeting the major criterion
Moderate or severe baseline thrombocytopenia (before PCI) (platelet count < 100 × 10^9^/l)	
Chronic bleeding diathesis	
Liver cirrhosis with portal hypertension	
	Long-term use of oral NSAIDs or steroids
Active malignancy (excluding non-melanoma skin cancer) within the past 12 months	
Previous spontaneous ICH (at any time) Previous traumatic ICH within the past 12 months Presence of a brain arteriovenous malformation Moderate or severe ischaemic stroke within the past 6 months	Any ischaemic stroke at any time not meeting the major criterion
Non-deferrable major surgery on DAPT	
Recent major surgery or major trauma within 30 days before PCI	

Patients are considered to be at high bleeding risk if at least one major or two minor criteria are met

*CKD* chronic kidney disease, *DAPT* dual antiplatelet therapy, *eGFR* estimated glomerular filtration rate, *ICH* intracranial haemorrhage, *NSAID* nonsteroidal anti-inflammatory drug

Thrombotic risk and (long-term) ischaemic risk are composed of both clinical (patient) characteristics and technical aspects related to the PCI or the coronary anatomy. High-risk features of stent-driven recurrent ischaemic events can be identified, such as prior stent thrombosis, treatment of bifurcation lesions or diffuse multivessel disease, especially in patients with diabetes. In addition, the DAPT score can be used, which has been validated in order to assess the benefit of DAPT continuation beyond 1 year after stenting [17].

As both bleeding and ischaemic risk are complex, we recommend assessing high bleeding risk and high ischaemic risk in a structured manner, using one of the above tools prior to discharge. In addition, the interventional cardiologist should take the leading role in highlighting any high-risk features of recurrent ischaemic events, related to the PCI procedure or coronary anatomy.

It is noteworthy that high bleeding risk and high ischaemic risk often overlap, as some risk factors (e.g. age, renal failure) pre-dispose to both bleeding and ischaemic risk. In patients with both high bleeding and ischaemic risk, prioritising the bleeding risk and thus abbreviating or de-escalating DAPT should generally be considered. Specifically, bleeding risk should be prioritised in frail older patients, in patients with anaemia or previous bleeding and in patients with active malignancy (all known strong predictors for bleeding).

Finally, the decision to continue DAPT beyond 1 year can be postponed to follow-up in the outpatient clinic. Patients who do not experience any bleeding events during the initial course of DAPT and have a high ischaemic risk should be considered for intensified antithrombotic therapy (DAPT continuation or aspirin plus very low-dose rivaroxaban) [18].

## Timing of invasive coronary angiography

### ST-elevation myocardial infarction

In the Netherlands, extensive efforts have been made to ensure proper pre-hospital diagnosis and treatment of STEMI. In this setting, immediate reperfusion therapy by pPCI should be performed within 120 min of the diagnosis. Therefore, we recommend that all patients presenting with symptoms suggestive of ACS and an electrocardiogram consistent with STEMI be transferred immediately to a PCI centre to undergo urgent invasive coronary angiography (ICA) and, in most cases, pPCI. The clinical benefit of an immediate invasive strategy in patients presenting within 12 h after the onset of symptoms is well established. In late presenters (> 12 h after symptom onset), the evidence for immediate ICA (± PCI) is less robust, although imaging studies have provided evidence of myocardial salvage if PCI is performed [19]. In late presenters (> 12 h and < 48 h), immediate ICA should be considered based on the extent of myocardium at risk, ongoing signs of ischaemia and/or haemodynamic or electrical instability. In patients presenting > 48 h after symptom onset who are clinically stable, PCI of an occluded infarct-related vessel is not recommended (class III, LoE A).

### Non-ST-elevation acute coronary syndrome

#### Immediate (< 2 h)

**Fig. 1:**
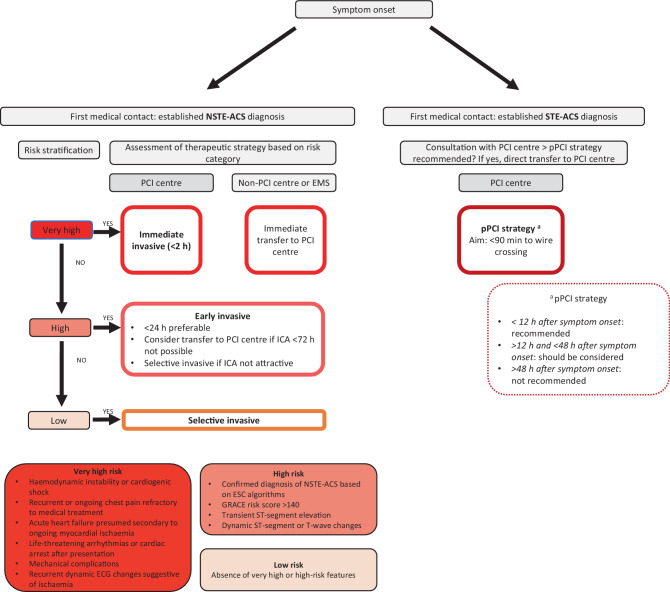
Invasive strategies according to symptom onset and stratified for acute coronary syndrome (*ACS*) diagnosis. *NSTE-ACS* non-ST-elevation acute coronary syndrome, *pPCI* primary percutaneous coronary intervention, *EMS* emergency medical service, *ICA* invasive coronary angiography, *ESC* European Society of Cardiology, *GRACE* Global Registry of Acute Coronary Events

Taking into account the poor prognosis if patients are left untreated, we endorse the unchanged recommendation in the 2023 ESC guidelines that an immediate invasive strategy is required in very high-risk patients. The presence of ≥ 1 very high-risk criterion justifies immediate ICA or transfer to a PCI centre (Fig. 1).

#### Early (< 24 h)

In line with our previous critical appraisal [20], the 2023 ESC guidelines have downgraded the recommendation to perform ICA < 24 h in patients with an established diagnosis of NSTE-ACS from class I, LoE A (2020) to class IIa, LoE A. Especially in cases of low to intermediate risk (Global Registry of Acute Coronary Events (GRACE) score < 140), there is limited evidence that an early invasive strategy improves clinical outcomes in patients with NSTE-ACS [21]. The main limitations to the interpretation of the RCTs are different study designs (particularly with regard to timing of delayed ICA) and the fact that time to ICA was generally based on randomisation time rather than onset of symptoms. Following the publication of the 2020 guidelines, only one new RCT investigating timing of ICA has been published. Although this trial tested immediate versus early ICA and therefore also has its limitations regarding comparison with other studies, no significant difference in clinical outcome was observed [22]. In an updated meta-analysis, which included a post hoc analysis of the impact of the GRACE risk score on all-cause mortality in the VERDICT trial, no clear advantage of an early strategy was demonstrated as regards mortality, except for patients with the highest risk (GRACE score > 140) [23].

Considering the afore-mentioned limited additional data, our recommendation for the timing of ICA in NSTE-ACS remains unchanged. We advise endeavouring for an early invasive strategy (< 24 h), specifically in patients with a GRACE risk score higher than 140. If this is not possible from a logistical perspective, a delayed invasive strategy (ICA within 72 h) is acceptable and safe.

### Invasive coronary angiography in resuscitated patients after cardiac arrest

In patients presenting with out-of-hospital cardiac arrest (OHCA) and persistent ST-segment elevation, recommendations are similar to those for STEMI without cardiac arrest. In OHCA without persistent ST-segment elevation, the ESC guidelines provide a revised recommendation regarding the timing of ICA. Immediate angiography is downgraded to class III, LoE A, based on the results of the COACT and TOMAHAWK trials [24, 25]. In both trials, a strategy of immediate versus delayed ICA did not affect all-cause mortality. Meanwhile, a class IIa (LoE C) recommendation was decided upon for immediate transfer to a cardiac arrest centre. This recommendation is based on retrospective data only, while the recently published ARREST trial showed no difference in mortality whether patients were transferred to a cardiac arrest centre or the nearest hospital [26]. Of note is that requirements for cardiac arrest centres included a set of 13 minimum criteria related to ICU care and availability of cardiothoracic facilities, such as immediate coronary angiography and mechanical circulatory support. As such, with regard to the Dutch healthcare system, these arrest centres are most similar to hospitals with cardiothoracic surgery.

Based on the evidence stated above, the Dutch ACS Working Group agrees with the recommendation not to perform ICA < 24 h in patients with OHCA without signs of STEMI. Furthermore, we advise that current regional arrangements for haemodynamically unstable patients without STEMI not be changed.

### Transfer to PCI centre

The 2020 recommendation regarding same-day transfer of all high-risk NSTE-ACS patients to a PCI centre has been rephrased and refers to ‘early inpatient transfer’. As postulated in our previous document, we advise that this recommendation be considered in the context of regional arrangements between PCI centres and referring hospitals. Performing ICA in a centre with PCI capabilities may avoid performing two invasive procedures in the same patient, while angiography in a non-PCI centre relieves the burden on ambulance services and PCI centres, as a substantial number of patients do not undergo PCI.

## Completeness and timing of revascularisation

Multivessel disease (MVD) is present in approximately 50% of all patients presenting with ACS. Recommendations regarding the extent and timing of revascularisation vary according to the clinical syndrome (NSTE-ACS versus STEMI) or setting, such as cardiogenic shock. In acute MI complicated by cardiogenic shock, where MVD is found in 85% of patients, a strategy of complete versus culprit-only revascularisation was associated with increased 30-day rates of death or renal replacement therapy in the randomised CULPRIT-SHOCK trial [27]. The 2023 guidelines therefore recommend PCI of the infarct-related artery (IRA) only in the acute setting (class I, LoE B) and staged PCI of non-IRA lesions (class II, LoE C) [1]. Nonetheless, there may be cases where multivessel PCI in the acute setting is reasonable, such as when there is uncertainty regarding the true culprit lesion in MVD.

The benefits of complete revascularisation have been clearly established in the setting of STEMI. A large-scale meta-analysis of 7542 patients from ten RCTs showed a significant reduction in cardiovascular mortality and MI with complete revascularisation as compared with culprit-only PCI [28]. Thus, complete revascularisation has a class I, LoE A recommendation. Uncertainty still exists regarding the optimal timing of revascularisation. The guidelines recommend performing either immediate revascularisation or staged within 45 days, i.e. during initial hospitalisation or after discharge (class I, LoE A). Until a clear benefit of any of these potential timing options has been established, it remains reasonable to decide on a timing option based on local infrastructure and the operator’s and patient’s preferences.

Moreover, it remains unknown whether physiology-guided complete revascularisation offers additional benefits over angiographic guidance in STEMI patients. Smaller studies have shown no difference in outcomes between physiology-guided versus angiography-guided PCI in the setting of complete revascularisation in STEMI [29]. Therefore, routine physiological guidance in this setting is not recommended at this time, and the COMPLETE-2 RCT (NCT05701358) with > 5100 patients is currently addressing this question. Furthermore, timing of revascularisation and the use of intracoronary physiology are investigated in the iModern trial [30], in which STEMI patients with residual non-culprit lesions are randomised to instantaneous wave-free-ratio-guided treatment of non-culprit lesions during the index procedure versus deferred cardiac-magnetic-resonance-guided management within 4 days to 6 weeks.

To date, no dedicated RCT has investigated the impact of complete versus culprit-only PCI in NSTE-ACS patients with MVD. Nonetheless, the 2023 guidelines include a class IIa, LoE C recommendation for complete revascularisation. In contrast to the recommendation in the setting of STEMI, functional invasive evaluation during the index procedure may be considered in NSTE-ACS (class IIb, LoE B). Moreover, in the BIOVASC trial, immediate complete revascularisation as compared with staged revascularisation was associated with a reduction in MI and unplanned ischaemia-driven revascularisation in the NSTE-ACS subgroup [31].

With regard to the use of intracoronary imaging to guide multivessel revascularisation in the setting of ACS, the 2023 guidelines do not include any recommendations due to a lack of randomised evidence.
